# Case Report: IgG multiple myeloma and chronic myeloid leukemia in a single patient

**DOI:** 10.12688/f1000research.24086.2

**Published:** 2020-09-23

**Authors:** Neeraja Swaminathan, Sorab Gupta, Claudia Dourado

**Affiliations:** 1 Department of Internal Medicine, Albert Einstein Medical Center, Philadelphia, PA, 19141, USA; 2 Department of Hematology, Albert Einstein Medical Center, Philadelphia, PA, 19141, USA

**Keywords:** multiple myeloma, MGUS, CML, chronic myeloid leukemia

## Abstract

A 58-year-old man presented with recurrence of chronic myeloid leukemia (CML) after complete molecular remission in the setting of non-compliance with imatinib. He was restarted on imatinib and was also noted to have IgG kappa monoclonal gammopathy of undetermined significance (MGUS). The patient re-achieved molecular remission after resumption of imatinib, but his MGUS progressed to smoldering myeloma and he was eventually diagnosed with multiple myeloma (MM) and initiated on treatment for MM with thalidomide, bortezomib and dexamethasone. He has responded well to treatment of the myeloma and continues concurrent maintenance imatinib treatment for CML and is being evaluated for bone marrow transplant. The association of two concurrent hematological malignancies, CML and MM, is very rare and has been infrequently reported in literature. The pathophysiology of this has not yet been fully understood. This case report reviews the various theories to explain this and discusses the potential challenges of simultaneous treatment of MM and CML.

## Introduction

Chronic myeloid leukemia (CML) is a clonal myeloproliferative disorder of pluripotent hematopoietic stem cells. It results from a translocation t (9;22) (q34q11) known as the Philadelphia chromosome creating a BCR-ABL fusion gene, which is transcribed into proteins with abnormal tyrosine kinase activity that drives abnormal white blood cell (WBC) proliferation ^1^. Multiple myeloma (MM) is a monoclonal disorder of plasma cells which have differentiated from lymphoid B cells. Therefore, the abnormal cell types in CML and MM are distinctly different. The instances of both MM and CML in the same patient occurring in a synchronous or metachronous manner are extremely rare. There are several factors that have been postulated to be related to this occurrence. These include age, gender, race, exposure to environmental carcinogens or radiation, epigenetic upregulation/downregulation, as a result of progression or treatment of one malignancy potentiating the development of malignant cells (which acquire antiapoptotic ability) and mechanisms to evade immune surveillance, chronic antigenic stimulation, genetic polymorphisms. At present there are multiple theories but insufficient data to make any definite conclusions about the mechanism of co-existence of CML and MM. With the advent of novel therapies and improving survival in patients with CML and MM, there is value in further investigation regarding the pathophysiology and clinical characteristics of such cases ^2, 3^. Additionally, the occurrence of more than one hematological malignancy in the same patient presents treatment challenges because it can lead to the possibilities of drug-drug interactions and medication toxicities. Analysis of the treatment protocols of these patients and their follow-up will be required to assess the risks and benefits of different treatment options. At this time, due to paucity of data, the management is tailored according to the patient’s individual risk factors and clinician’s judgement.

This is a rare case of the occurrence of IgG MM and CML in a single patient and reviews the management of these diseases.

## Case report

A 58-year-old man with history of CML presented in December 2015 for re-establishment of care after being lost to follow-up. He was first diagnosed in December 2007 with CML and was treated with imatinib (400mg daily). He achieved complete molecular remission but was unfortunately lost to follow-up after 2012. The patient stated that he had stopped taking imatinib in September 2015 due to family issues and stressors. When he presented in December 2015, he had no specific complaints. He was noted to have mild pallor on exam, but no lymphadenopathy or hepatosplenomegaly. Labs were significant for leukocytosis and blasts noted on peripheral smear. BCR-ABL FISH/PCR was also positive indicating relapse of CML and the transcripts at the time of diagnosis were typical. His peripheral smear showed only rare blasts (< 10 %) and the patient appeared to be in the chronic phase of CML. A bone marrow biopsy was not repeated at this time.

The patient was re-initiated on treatment with imatinib 400mg daily. Within 2 weeks of resuming treatment he was noted to have an improvement in WBC count. However, he was incidentally detected to have an elevated total protein level. In view of this serum protein, electrophoresis was ordered and the patient was found to have an IgG kappa monoclonal gammopathy of undetermined significance (MGUS). This diagnosis was established because the total M protein spike was <3g/dL, bone marrow biopsy showed < 10% plasma cells and the patient had no evidence of end organ damage. Skeletal survey was done which showed no lytic lesions. Imatinib was continued for CML and he was monitored closely for progression of plasma cell dyscrasia.

With regard to the patient’s CML, he achieved molecular remission in November 2016 with 3 log reduction in the 3 tested transcripts b2a2, b3a2, e1a2 and without detectable Philadelphia chromosome. However, he had a steady increase in the paraprotein level from December 2015 to April 2019 without any symptoms. He did not develop myeloma defining events such as hypercalcemia (>1mg/dL over the upper limit of normal OR >11mg/dL), renal insufficiency (creatinine clearance <40mL/min or serum creatinine >2mg/dL), anemia (Hemoglobin > 2g/dL below lower limit of normal or <10g/dL) or bony pain/lytic lesions (on skeletal radiography/CT/PET). These are together referred to as the CRAB phenomenon. His serum free light chain ratio remained at <100mg/L.

In May 2019, the patient had further rise in creatinine and paraprotein level and had an increase in serum free light chain ratio. Therefore, he underwent a PET CT in June 2019. This showed increased uptake in the left 4 ^th^ rib, left and right ischium, and a lesion in the second lumbar (L2) vertebra. He also underwent a bone marrow biopsy in July 2019, which showed 50% plasma cells expressing CD 38, CD 138, dim/partial CD 117, CD 56 and kappa light chain restriction. No BCR ABL gene rearrangement was noted. Thus, it confirmed the diagnosis of MM.

MM interphase fluorescence in situ hybridization (FISH) panel analysis of CD138+ enriched plasma cells was positive for three CCND1 signals consistent with trisomy 11 and for extra signals for chromosomes 7, 9 and 15. There were no cells with FGFR3-IGH, CCND1-IGH, or IGH-MAF fusions. Results for 1p/1q, 13q and TP53 were normal. Extra signals for probes targeting the chromosomes reported above suggest the presence of a hyperdiploid clone. Thus, the patient’s cytogenetic testing was negative for high risk factors.

**Figure 1.**
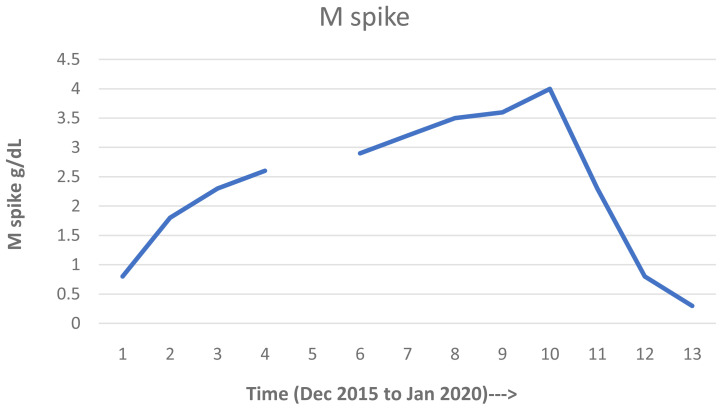
Graphical trend of M spike.

**Figure 2.**
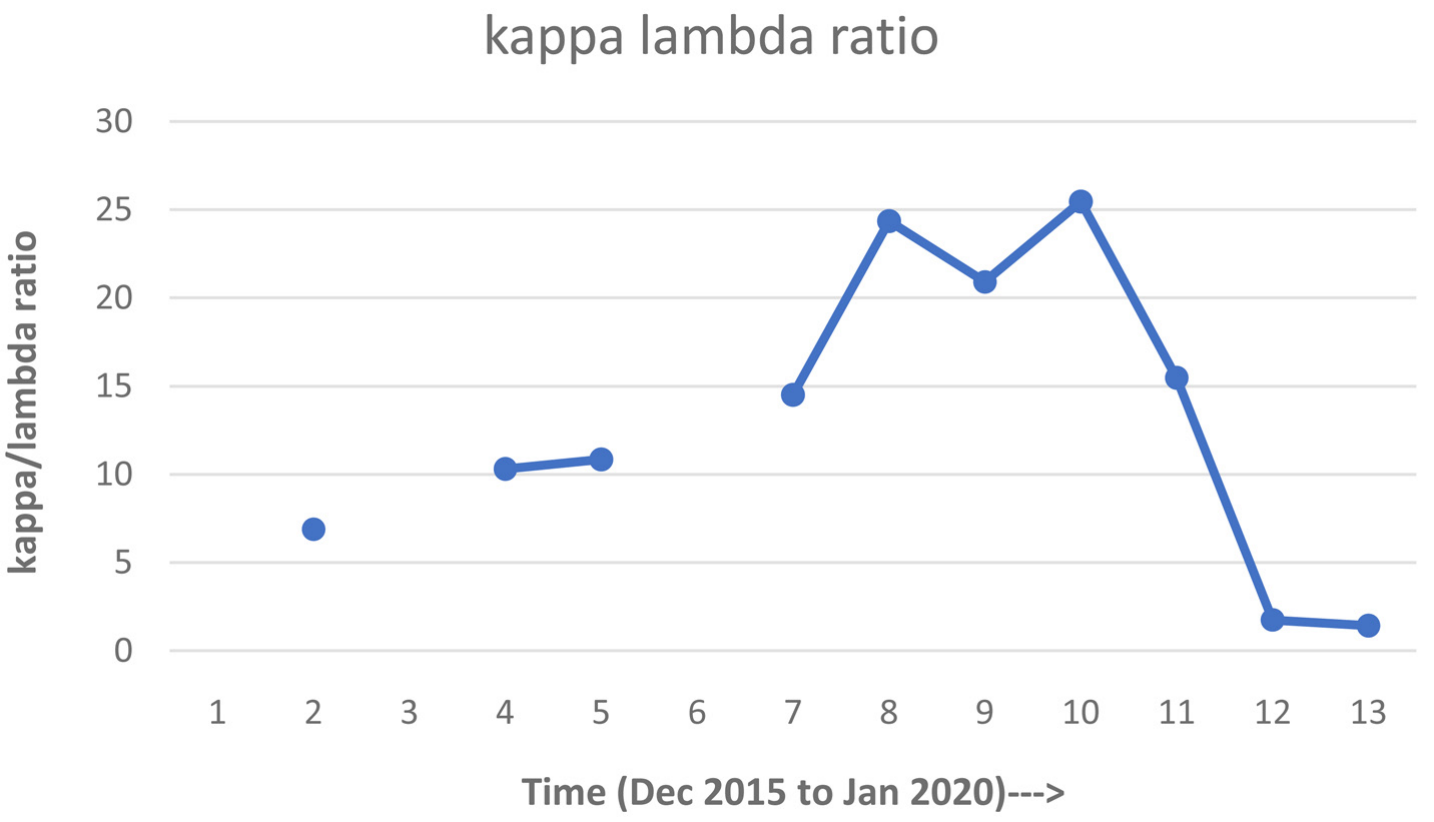
Graphical trend of kappa/lambda ratio.

**Figure 3.**
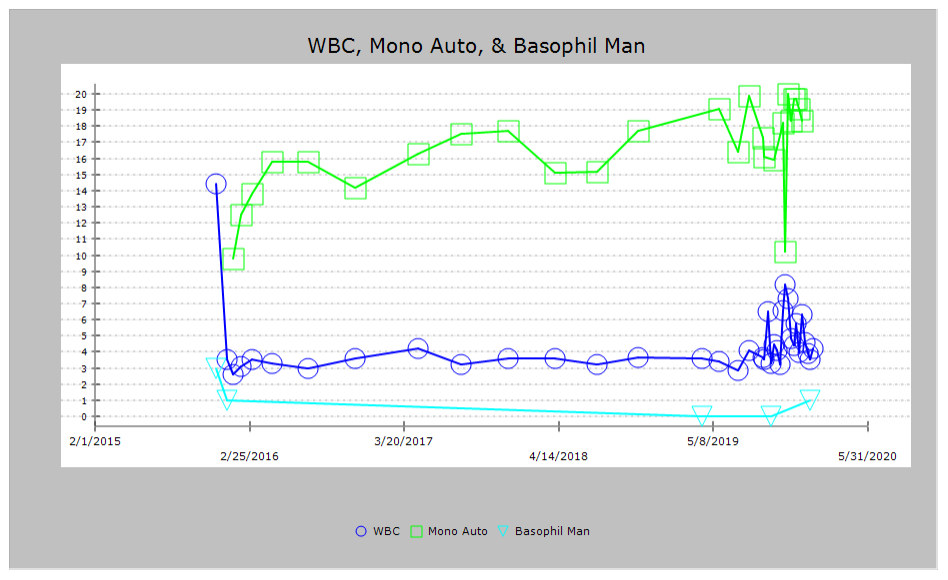
Graphical trend of white blood cell (WBC) count, lymphocytes and basophils.

The trends of serum protein electrophoresis are seen in Table 1, and Figure 1 and Figure 2. Trends of WBC count, hemoglobin, platelet count, and creatinine are seen in Figure 3– Figure 6, respectively.

**Table 1.** Serum electrophoresis results.

	A/G ratio	alpha 1 globulin	alpha 2 globulin	beta globulin	gamma globulin	M spike	kappa lambda ratio	IgG	IgA	IgM	beta 2 microglobin	Lactate dehydrogenase
**Dec-15**	1.1	0.2	0.6	0.8	2.1	0.8						
**Nov-16**	0.9	0.2	0.5	0.7	4.1	1.8	6.89	3103	<5	27		
**Apr-17**	0.8	0.2	0.6	0.8	3.2	2.3		3239	<5	19		
**Aug-17**	0.8	0.1	0.5	0.8	3.3	2.6	10.3	3770	<5	17		233
**Dec-17**							10.84	3826	<5	16		
**Jul-18**	0.7	0.2	0.6	0.9	3.7	2.9		3931	<5	13		
**Oct-18**	0.8	0.1	0.5	0.7	3.7	3.2	14.5	3871	<5	14		
**Apr-19**	0.7	0.2	0.6	0.8	4.1	3.5	24.35					
**May-19**	0.7	0.2	0.6	0.8	4.1	3.6	20.91	4456	<5	11	4.2	
**Sep-19**	0.6	0.2	0.7	0.8	4.5	4	25.47	5367	<5	9	4	169
**Oct-19**	0.8	0.2	0.6	0.7	2.5	2.3	15.48					175
**Nov-19**	1.1	0.2	0.7	0.8	1.1	0.8	1.75	1176	<5	26		377
**Dec-19**	1.4	0.2	0.6	0.8	0.8	0.3	1.43					
**Jan-20**	1.6	0.2	0.6	0.7	0.7	faint	1.35					

Abbreviations used: A/G ratio- albumin/globulin ratio, M spike- monoclonal spike, Ig- immunoglobulin, LDH-lactate dehydrogenase.

**Figure 4.**
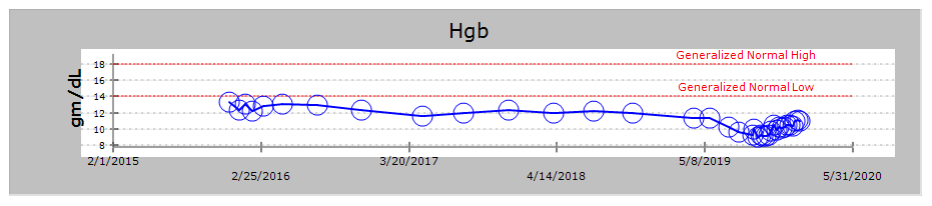
Graphical trend of hemoglobin.

**Figure 5.**
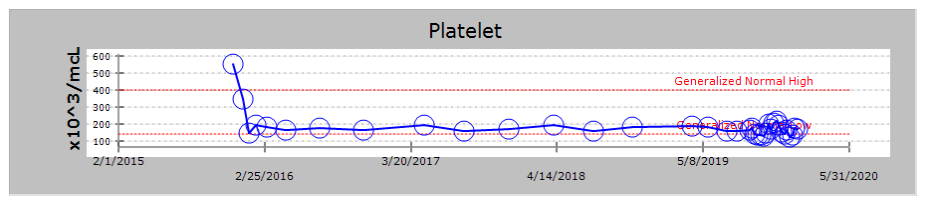
Graphical trend of platelet count.

**Figure 6.**
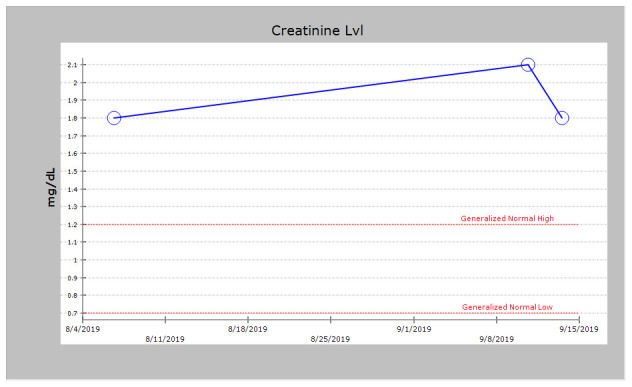
Graphical trend of creatinine.

The patient was started on treatment for ISS stage II standard risk myeloma with thalidomide (50mg/day daily for 21 days followed by 7 days off), weekly bortezomib (1.3mg/m ^2^) and dexamethasone (40mg/day once a week) in September 2019. This regimen was preferred over the VRd (lenalidomide, bortezomib and dexamethasone) due to the concern for increased risk of pancytopenia with concurrent use of imatinib and lenalidomide.

After cycle 2 of the myeloma treatment, this patient developed a morbilliform pruritic rash over trunk and bilateral upper extremities which has been described as a side effect of thalidomide. He was managed with a short course of steroids. After cycle 5, he had recurrence of above described rash and also developed conjunctival injection bilaterally. He was seen by ophthalmology and diagnosed to have meibomian gland dysfunction. Due to the rash and ocular symptoms, there were delays in his myeloma treatment but these were not dose-limiting toxicities. His symptoms improved with supportive care (oral steroids and steroid eye drops) and resolved completely. Thus far he has completed 6 cycles of treatment for myeloma with concurrent imatinib and has been referred to a transplant center to assess his eligibility for the same.

## Discussion

The occurrence of CML and MM together is very rare. There are multiple explanations that have been suggested for co-existence of more than one hematological malignancy. One of the theories is that there is a common progenitor stem cell. The Philadelphia chromosome is observed not only in granulocytes but also in cells of the monocytic, erythroid, megakaryocytic and lymphoid series. This finding supports the concept of a common pluripotent progenitor cell. The transformation to lymphoid cells in the blast phase of the CML also suggests a relationship between the myeloid and lymphoid lineage ^4^.

The role of imatinib in promoting development of MM is debatable. There is some evidence from Pandiella *et al*. ^5^ that imatinib inhibits MM cell proliferation *in vitro*. On the contrary, it has been reported that imatinib has a stimulatory effect on MM cells through activation of Erk1 and Erk2 mitogen activated protein kinases (MAP kinases). There have also been reports of MM developing in non CML patients such as those with gastrointestinal stromal tumors treated with imatinib ^6^ Carulli *et al*. ^7^ looked at the possible interference of imatinib with plasma cell phenotype. Their study looked at 30 patients and found that 70% of these patients had an abnormal plasma cell phenotype, which they defined as plasma cells which lack CD 19. Some of these cells showed additional aberrations such as expression of CD 56. Although this cannot establish a causal relationship between imatinib and MM, it merits further investigation.

Imatinib mesylate is a tyrosine kinase inhibitor, which is the standard of care for CML as a first line agent. It has activity against different genes involved in cellular transformation such as BCR-ABL1, c-KIT, PGFR-α and β and Jak 2. Imatinib in CML acts by competing with ATP to bind to the BCR ABL1 tyrosine kinase and thereby inhibiting the WBC proliferation that it effects ^7^.

Treatment of MM is based on whether the patient has standard or high-risk disease, which in turn is determined by cytogenetic analysis. High risk features constitute t (14;16), t (4;20), del17p13, t (4;14) and 1q gain. Additionally, patients should be assessed for eligibility to receive an autologous stem cell transplant. In standard risk patients such as ours the standard first line treatment is RVd regimen, which comprises lenalidomide, bortezomib and dexamethasone. Post induction therapy, if patients are eligible for hematopoietic cell transplant (HCT), they may choose either an early HCT or delayed HCT strategy. Autologous HCT is the mainstay, although allogenic HCT is still largely investigational. Post-transplant patients need maintenance therapy as well. Transplant in eligible patients receive either a two/three drug induction regimen followed by maintenance therapy. Melphalan, cyclophosphamide and thalidomide are also part of first line treatment options for myeloma. In patients with relapse, several of the newer agents are being used, which include the new proteasome inhibitor (carfilzomib), immunomodulatory drugs (like pomalidomide), inhibitors of NF-κB, MAPK and AKT, histone deacetylase inhibitors (like vorinostat and panobinostat), and monoclonal antibodies (such as daratumumab, elotuzumab and siltuximab) ^8^.

Due to the rarity of coexistence of more than one hematological malignancy in the same patient, we do not have robust data on treatment regimens. Current treatment is based on factoring in the patient’s individual risk factors and the physician’s judgement and experience. Even though there is concern for imatinib being associated with MGUS and myeloma, co-administration of imatinib with myeloma treatment seems to be reasonable. Myeloma treatment both for high risk and standard risk group patients involves a proteasome inhibitor, such as bortezomib. Both bortezomib and imatinib are metabolized by microsomal enzyme CYP3A4. However, imatinib is a potent inhibitor of CYP3A4, while bortezomib is only a weaker inhibitor. Reduction of bortezomib dosing to once weekly instead of twice seems to be associated with less adverse effects when used in conjunction with imatinib ^9^.

Additionally, bisphosphonates, which are used as supportive treatment in myeloma, have also been shown to inhibit CML cell lines and induce apoptosis synergistically with imatinib through the inhibition of prenylation of Ras and Ras-related proteins ^10^

This case report presents an experience with a CML patient who over time progressed to develop the entire spectrum of myeloma, starting with MGUS followed by smoldering myeloma and ultimately MM requiring treatment. At this point it is difficult to say if this association is coincidental or related to some other mechanism and this is a subject that requires further research.

## Conclusion

The most essential take-home points from this case are as follows:

1. MM can be diagnosed in patients with one of the following: >10% plasma cells in bone marrow OR biopsy proven bony/extramedullary plasmacytoma and any one of the CRAB phenomenon OR any of the following- >/= 60% plasma cells in bone marrow OR serum free light chain ratio >/= 100 OR >1 focal lesion on MRI studies.
2. Both MGUS and smoldering myeloma are characterized by an absence of myeloma defining events. In MGUS: paraprotein level <3g/dL, bone marrow plasma cells <10%; while in smoldering myeloma: paraprotein level >3g/dL or urinary monoclonal protein >/= 500mg in 24 hours and/or clonal bone marrow plasma cells 10 to 60%
3. Co-existence of CML and MM is very rare. Etiology is probably multifactorial but there is a possibility that this is because CML and MM share a common pluripotent progenitor stem cell which can differentiate into both lymphoid and myeloid lines.
4. There is concern that the imatinib may lead to alteration of the plasma cell phenotype and it may be worthwhile to monitor serum electrophoresis and protein levels in patients who have received imatinib treatment.
5. Co-administration of bortezomib and imatinib is feasible. Since CYP3A4 is important for metabolism of both drugs, administration of bortezomib as weekly instead of twice weekly may help to reduce adverse effects of bortezomib.

## Data availability

All data underlying the results are available as part of the article and no additional source data are required.

## Consent

Written informed consent for the publication of the case report and any associated images was obtained from the patient.
