# Supplementary material for: Case Report: IgG multiple myeloma and chronic myeloid leukemia in a single patient
Source: F1000Res. 2020 Sep 23;9:488. Originally published 2020 Jun 1. [Version 2] doi: 10.12688/f1000research.24086.2 (PMC7527863; doi:10.12688/f1000research.24086.2)
Supplement: Peer review history

# Peer review: Reviewer response for version 2

**DOI:** 10.5256/f1000research.28094.r71881

**Published:** 2020-09-30

**Authors:** Mahesh Swaminathan

**Affiliations:** 1 Roswell Park Comprehensive Cancer Center, Buffalo, NY, USA

No further comments.

Are enough details provided of any physical examination and diagnostic tests, treatment given and outcomes?

Yes

Is the case presented with sufficient detail to be useful for other practitioners?

Partly

Is sufficient discussion included of the importance of the findings and their relevance to future understanding of disease processes, diagnosis or treatment?

Yes

Is the background of the case’s history and progression described in sufficient detail?

Yes

Reviewer Expertise:

Myeloid leukemias, clinical trials, Genetic syndromes in leukemia.

I confirm that I have read this submission and believe that I have an appropriate level of expertise to confirm that it is of an acceptable scientific standard.

# Peer review: Reviewer response for version 1

**DOI:** 10.5256/f1000research.26569.r65599

**Published:** 2020-07-09

**Authors:** Pritish K. Bhattacharyya

**Affiliations:** 1 Department of Pathology, Hackensack University Medical Center, Hackensack, NJ, USA; 2 Rutgers New Jersey Medical School, Newark, NJ, USA

This is an uncommon case presentation of two apparently unrelated hematopoietic malignancies.

 Association of Multiple myeloma following CLL or other low grade B cell lymphoma may be related to terminal changes of B cell to Plasma cells (Jaffe *et al.*, NIH)

 We had published a similar case in 2014 in a patient of CML with t9:22) along with JAK2 mutation and Myeloma (Maerki *et al.*, 2014 ^1^). We suggested another potential theory of multiple simultaneous malignancies includes the sharing of a common malignant pluripotent progenitor stem cell which may allow further transformation of both lymphoid and myeloid differentiations.

 It has been proposed that plasma cell myeloma and CML may evolve from the same hematopoietic stem cell [1, 8, 12]. There have been multiple accounts in which plasma cell neoplasms, including plasma cell leukemia and multiple myeloma, have been seen in coexistence with or arising in the background of CML [37–39]. It is suggested that the simultaneous occurrence of CML and multiple myeloma is analogous to acute lymphoblastic leukemia arising in the blastic phase of CML [5, 6, 8, 12, 15]. This reveals the capability of CML in differentiating into either myeloid lineage or lymphoid lineage. Furthermore, the Philadelphia chromosome (Ph) has been linked to increased cell survival, proliferation, and malignant transformation [1, 40] and has been found in all hematopoietic cell lineages including erythropoietic cells, megakaryocytes, macrophages, and B-lymphocytes [1,12, 41].

Are enough details provided of any physical examination and diagnostic tests, treatment given and outcomes?

Yes

Is the case presented with sufficient detail to be useful for other practitioners?

Partly

Is sufficient discussion included of the importance of the findings and their relevance to future understanding of disease processes, diagnosis or treatment?

Yes

Is the background of the case’s history and progression described in sufficient detail?

Yes

Reviewer Expertise:

Leukemia, Lymphoma, Myeloma with molecular genetic applications

I confirm that I have read this submission and believe that I have an appropriate level of expertise to confirm that it is of an acceptable scientific standard.

## References

[1] : Unusual case of simultaneous presentation of plasma cell myeloma, chronic myelogenous leukemia, and a jak2 positive myeloproliferative disorder. *Case Rep Hematol*.2014;2014: 10.1155/2014/738428 738428 10.1155/2014/738428 25386371PMC4214051

# Peer review: Reviewer response for version 1

**DOI:** 10.5256/f1000research.26569.r64202

**Published:** 2020-06-08

**Authors:** Mahesh Swaminathan

**Affiliations:** 1 Roswell Park Comprehensive Cancer Center, Buffalo, NY, USA

In this case report, the authors have discussed a patient with chronic myeloid leukemia and metachronous IgG multiple myeloma. The clinical presentation and disease course are well described and discussed. 

 Adding the following details would add more clarity to the case and help readers:

1. Please mention the transcripts at diagnosis (as knowing typical versus atypical transcripts is important for follow up, the latter might not be detectable in routine PCR and warrants RT-PCR).
2. At CML disease relapse, please indicate if the patient was still in the chronic phase. Was there a bone marrow biopsy done at that time, if so what was the percentage of plasma cells. 
3. As the diagnosis of MGUS requires BMBx? Please indicate if the clonal plasma cells were <10% facilitating the diagnosis of MGUS.
4. Please indicate if the x-axis in Figure 1-2 denotes months?
5. It will be interesting to know the most common Grade >3 AEs experienced by the patient (to know if there were any specific side effects that were more common with concomitant imatinib and myeloma therapy.

Are enough details provided of any physical examination and diagnostic tests, treatment given and outcomes?

Yes

Is the case presented with sufficient detail to be useful for other practitioners?

Partly

Is sufficient discussion included of the importance of the findings and their relevance to future understanding of disease processes, diagnosis or treatment?

Yes

Is the background of the case’s history and progression described in sufficient detail?

Yes

Reviewer Expertise:

Myeloid leukemias, clinical trials, Genetic syndromes in leukemia.

I confirm that I have read this submission and believe that I have an appropriate level of expertise to confirm that it is of an acceptable scientific standard, however I have significant reservations, as outlined above.

## References

[1] : International Myeloma Working Group updated criteria for the diagnosis of multiple myeloma. *The Lancet Oncology*.2014;15(12) : 10.1016/S1470-2045(14)70442-5 e538-e548 10.1016/S1470-2045(14)70442-5 25439696

# Response

**Published:** 2020-06-13

**Authors:** Neeraja Swaminathan

**Affiliations:** Albert Einstein Medical Center, USA

Thank you for the feedback and comments provided. 

 

1. Yes, the transcripts at the time of diagnosis were also typical.
2. At the time of relapse, the patient was in the chronic phase. Only rare blasts were seen on peripheral blood smear. There were fewer than 10% blast cells in the blood and a bone marrow biopsy was not performed.
3. When patient was noted to have an abnormal M-protein spike on serum electrophoresis, a bone marrow biopsy was repeated which showed < 10% plasma cells.  The total M protein spike was <3g/dL and the patient had no evidence of end organ damage. All of this helped establish the diagnosis of MGUS and he was monitored clinically and with labs until May-June 2019 when he developed an increase in creatinine and eventually had a bone marrow biopsy which confirmed the diagnosis of multiple myeloma.
4. Yes, the x-axis in figures 1 and 2 denotes time in months.
5. This patient developed a morbilliform pruritic rash over trunk and bilateral upper extremities which has been described as a side effect of thalidomide. This occurred after cycle 2 and was managed with short course of steroids. After cycle 5, he had recurrence of above described rash and also developed conjunctival injection bilaterally. He was seen by ophthalmology and diagnosed to have meibomian gland dysfunction. In view of these symptoms, there was a delay in his myeloma treatment but his symptoms improved with supportive care (oral steroids and steroid eye drops) and resolved completely. He has now completed 6 cycles of treatment for myeloma and is being evaluated for bone marrow transplant. He has had no other side effects. 

# Response

**Published:** 2020-07-14

**Authors:** Mahesh Swaminathan

**Affiliations:** Roswell Park Cancer Institute, USA

Thanks for updating.

 Please make necessary inclusions of those comments in the manuscript.
